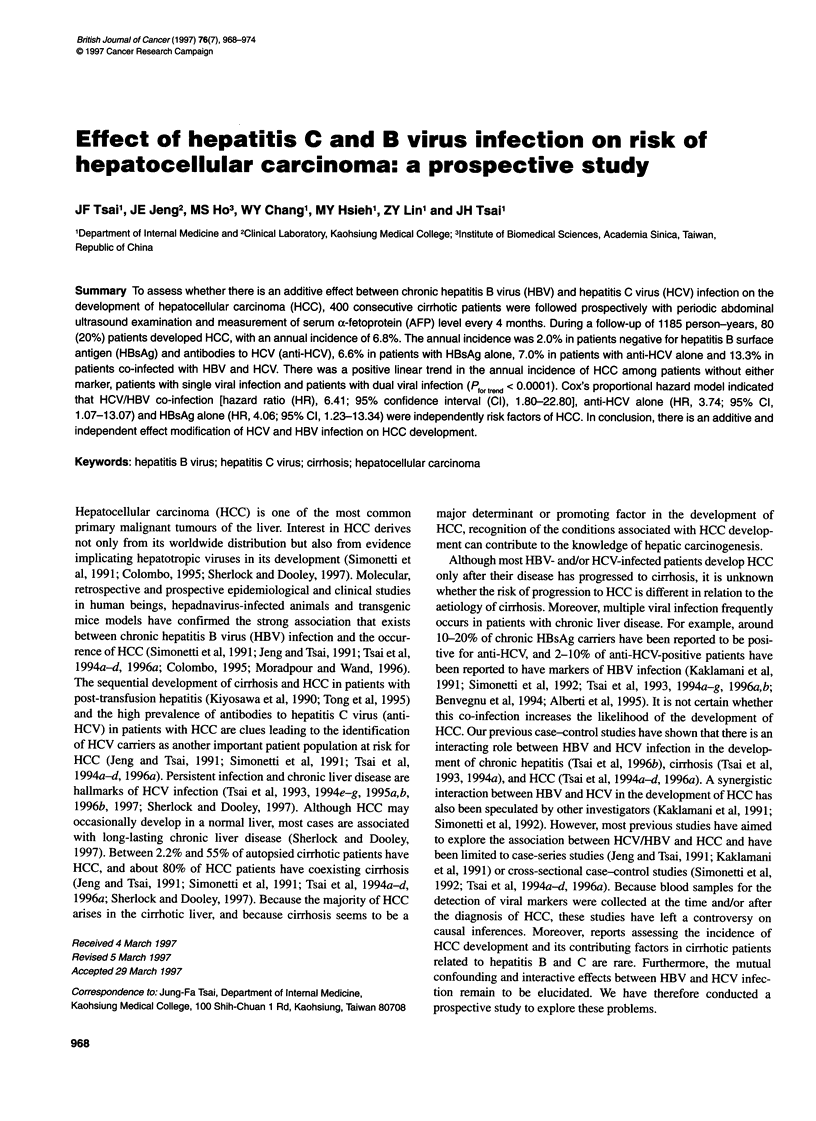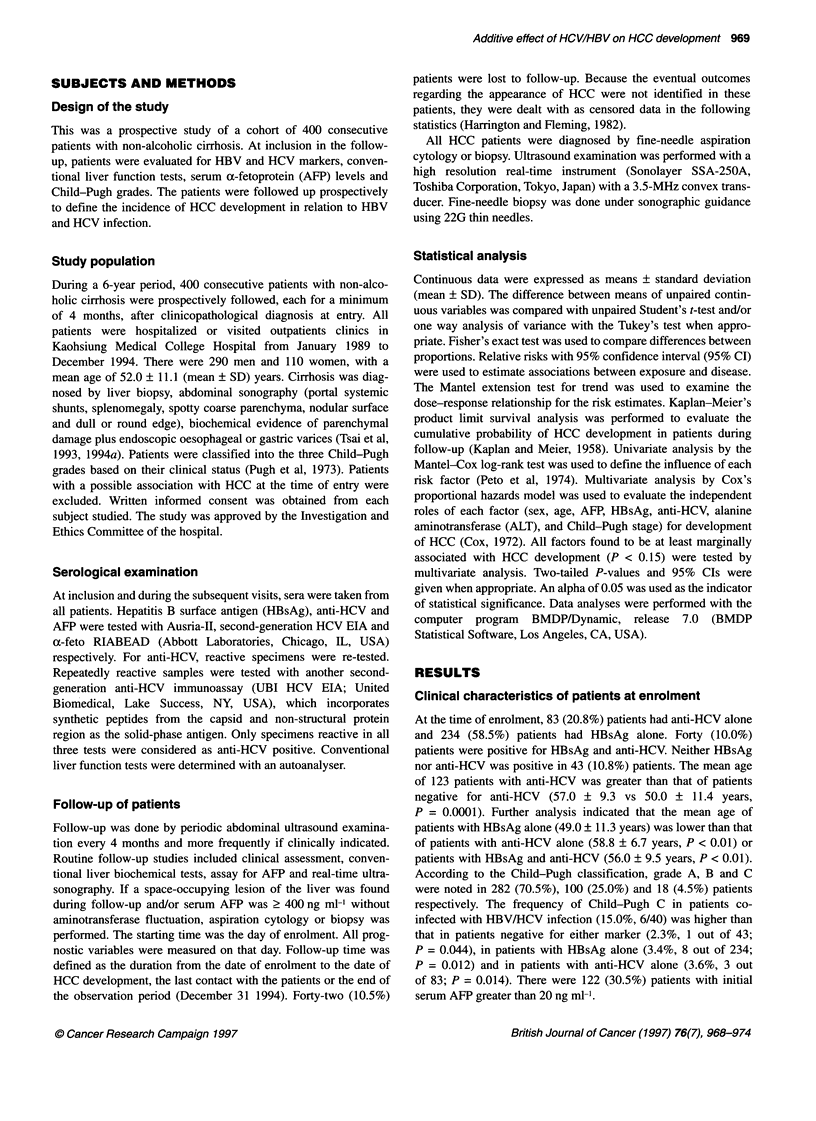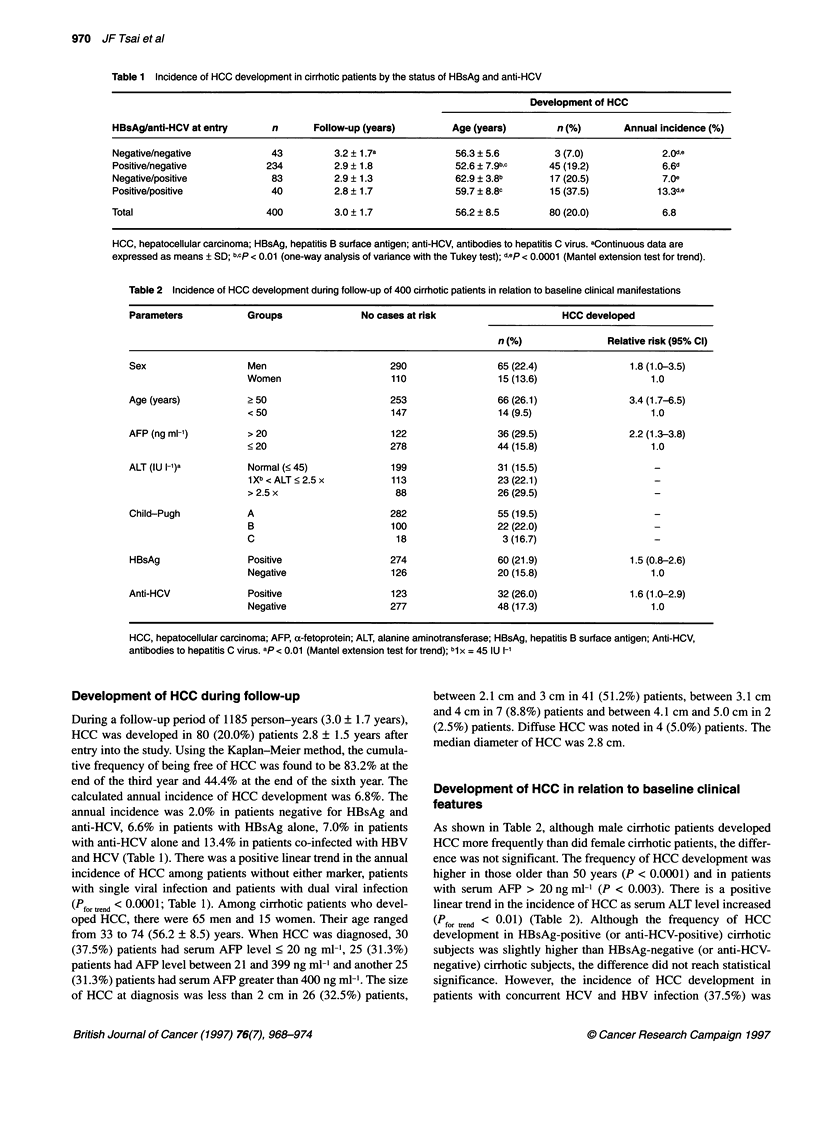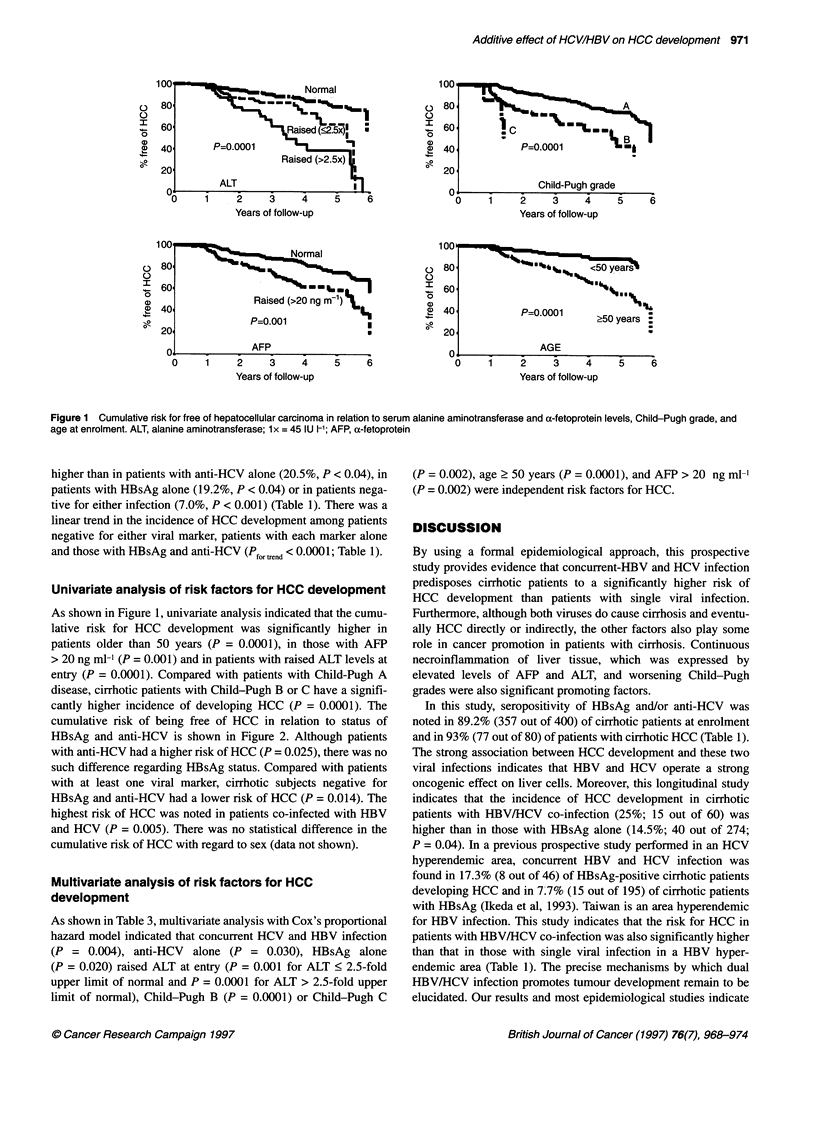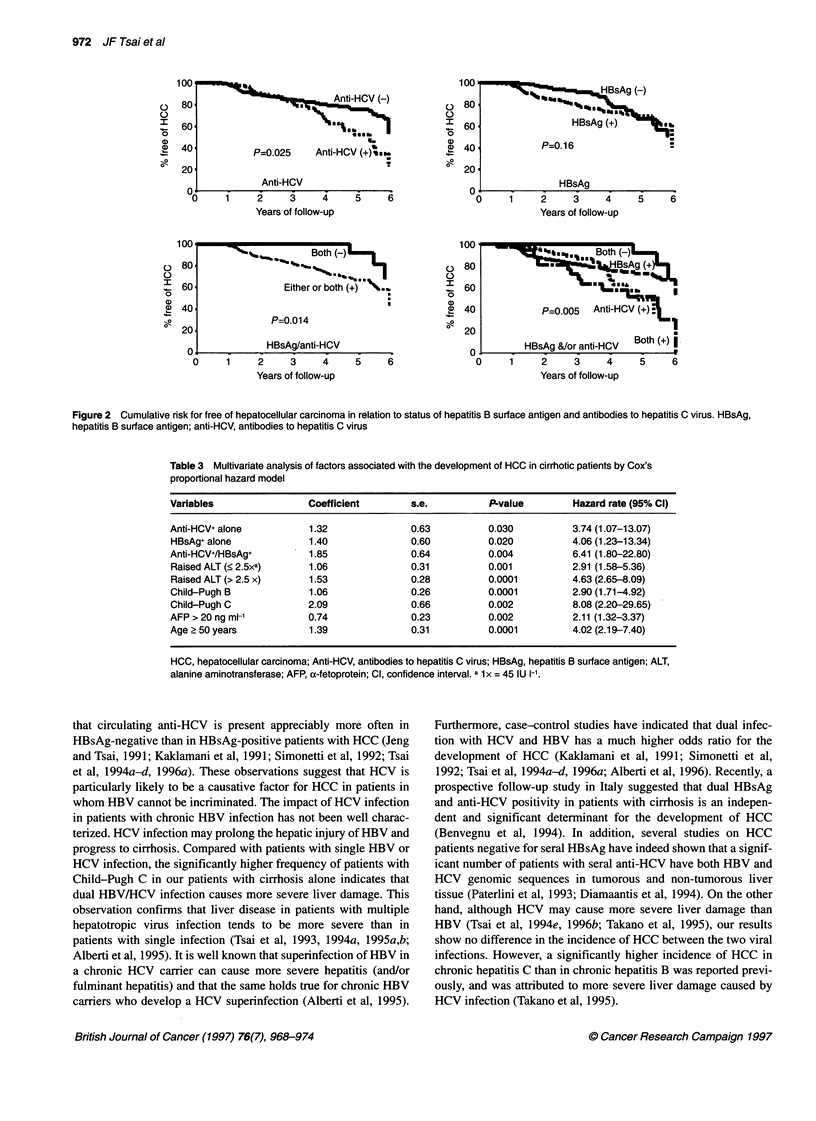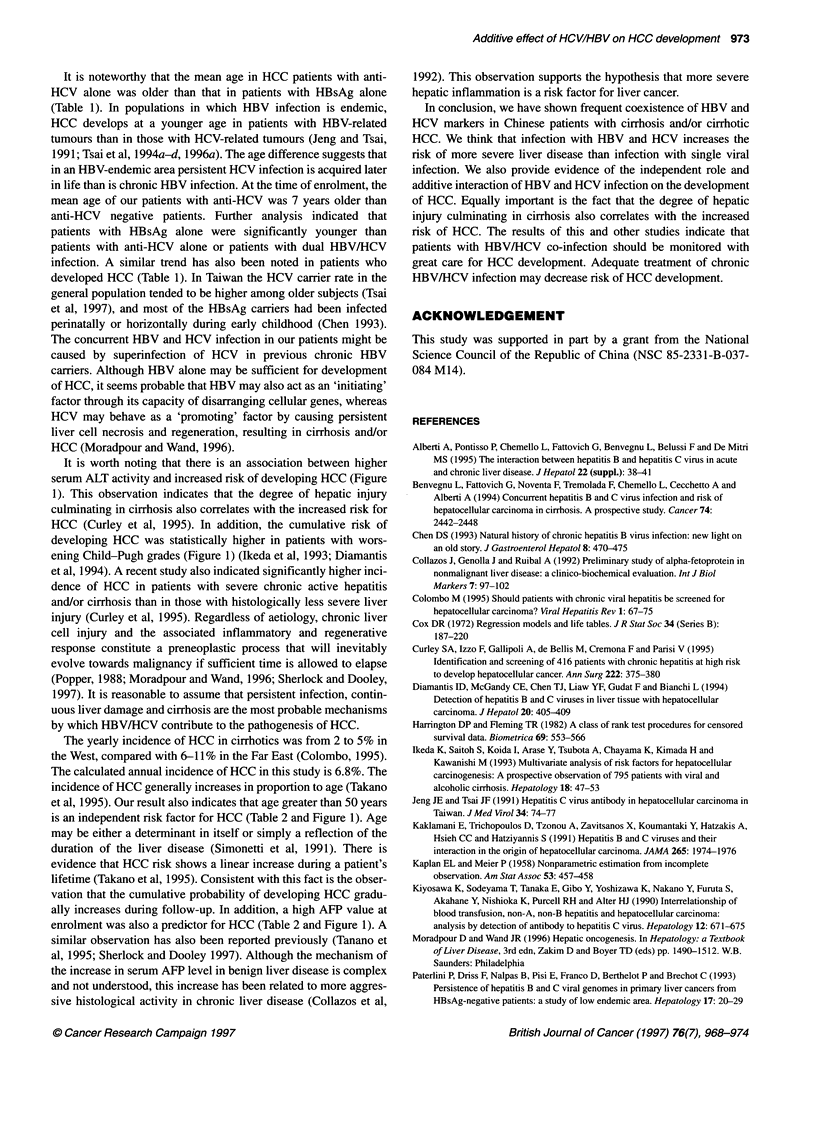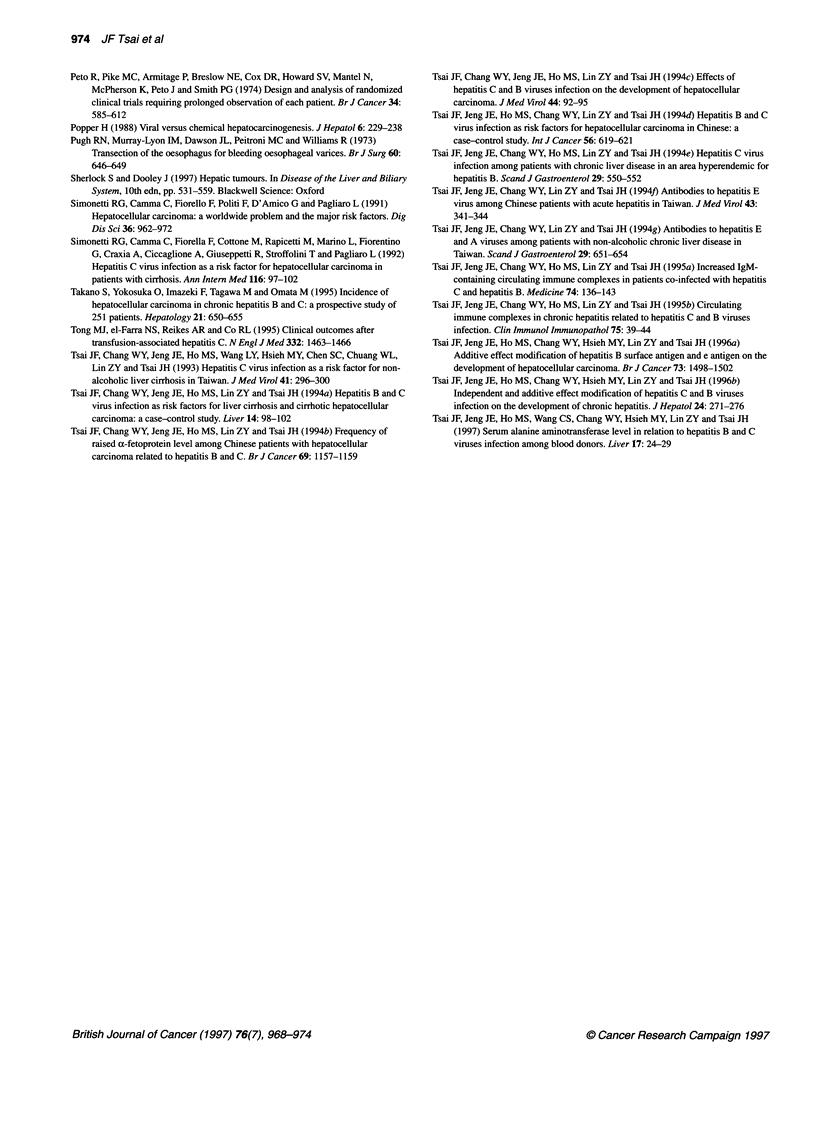# Effect of hepatitis C and B virus infection on risk of hepatocellular carcinoma: a prospective study.

**DOI:** 10.1038/bjc.1997.493

**Published:** 1997

**Authors:** J. F. Tsai, J. E. Jeng, M. S. Ho, W. Y. Chang, M. Y. Hsieh, Z. Y. Lin, J. H. Tsai

**Affiliations:** Department of Internal Medicine, Kaohsiung Medical College, Taiwan, Republic of China.

## Abstract

To assess whether there is an additive effect between chronic hepatitis B virus (HBV) and hepatitis C virus (HCV) infection on the development of hepatocellular carcinoma (HCC), 400 consecutive cirrhotic patients were followed prospectively with periodic abdominal ultrasound examination and measurement of serum alpha-fetoprotein (AFP) level every 4 months. During a follow-up of 1185 person-years, 80 (20%) patients developed HCC, with an annual incidence of 6.8%. The annual incidence was 2.0% in patients negative for hepatitis B surface antigen (HBsAg) and antibodies to HCV (anti-HCV), 6.6% in patients with HBsAg alone, 7.0% in patients with anti-HCV alone and 13.3% in patients co-infected with HBV and HCV. There was a positive linear trend in the annual incidence of HCC among patients without either marker, patients with single viral infection and patients with dual viral infection (P[for trend] < 0.0001). Cox's proportional hazard model indicated that HCV/HBV co-infection [hazard ratio (HR), 6.41; 95% confidence interval (CI), 1.80-22.80], anti-HCV alone (HR, 3.74; 95% CI, 1.07-13.07) and HBsAg alone (HR, 4.06; 95% CI, 1.23-13.34) were independently risk factors of HCC. In conclusion, there is an additive and independent effect modification of HCV and HBV infection on HCC development.


British Journal of Cancer (1997) 76(7), 968–974
© 1997 Cancer Research Campaign

### Effect of hepatitis C and B virus infection on risk of hepatocellular carcinoma: a prospective study

**JF Tsai[1], JE Jeng[2], MS Ho[3], WY Chang[1], MY Hsieh[1], ZY Lin[1] and JH Tsai[1]**

[1]Department of Internal Medicine and [2]Clinical Laboratory, Kaohsiung Medical College; [3]Institute of Biomedical Sciences, Academia Sinica, Taiwan, Republic of China

**Summary** To assess whether there is an additive effect between chronic hepatitis B virus (HBV) and hepatitis C virus (HCV) infection on the development of hepatocellular carcinoma (HCC), 400 consecutive cirrhotic patients were followed prospectively with periodic abdominal ultrasound examination and measurement of serum α-fetoprotein (AFP) level every 4 months. During a follow-up of 1185 person–years, 80 (20%) patients developed HCC, with an annual incidence of 6.8%. The annual incidence was 2.0% in patients negative for hepatitis B surface antigen (HBsAg) and antibodies to HCV (anti-HCV), 6.6% in patients with HBsAg alone, 7.0% in patients with anti-HCV alone and 13.3% in patients co-infected with HBV and HCV. There was a positive linear trend in the annual incidence of HCC among patients without either marker, patients with single viral infection and patients with dual viral infection ($P_{\text{for trend}} < 0.0001$). Cox's proportional hazard model indicated that HCV/HBV co-infection [hazard ratio (HR), 6.41; 95% confidence interval (CI), 1.80–22.80], anti-HCV alone (HR, 3.74; 95% CI, 1.07–13.07) and HBsAg alone (HR, 4.06; 95% CI, 1.23–13.34) were independently risk factors of HCC. In conclusion, there is an additive and independent effect modification of HCV and HBV infection on HCC development.

**Keywords**: hepatitis B virus; hepatitis C virus; cirrhosis; hepatocellular carcinoma

Hepatocellular carcinoma (HCC) is one of the most common primary malignant tumours of the liver. Interest in HCC derives not only from its worldwide distribution but also from evidence implicating hepatotropic viruses in its development (Simonetti et al, 1991; Colombo, 1995; Sherlock and Dooley, 1997). Molecular, retrospective and prospective epidemiological and clinical studies in human beings, hepadnavirus-infected animals and transgenic mice models have confirmed the strong association that exists between chronic hepatitis B virus (HBV) infection and the occurrence of HCC (Simonetti et al, 1991; Jeng and Tsai, 1991; Tsai et al, 1994*a–d*, 1996*a*; Colombo, 1995; Moradpour and Wand, 1996). The sequential development of cirrhosis and HCC in patients with post-transfusion hepatitis (Kiyosawa et al, 1990; Tong et al, 1995) and the high prevalence of antibodies to hepatitis C virus (anti-HCV) in patients with HCC are clues leading to the identification of HCV carriers as another important patient population at risk for HCC (Jeng and Tsai, 1991; Simonetti et al, 1991; Tsai et al, 1994*a–d*, 1996*a*). Persistent infection and chronic liver disease are hallmarks of HCV infection (Tsai et al, 1993, 1994*e–g*, 1995*a,b*, 1996*b*, 1997; Sherlock and Dooley, 1997). Although HCC may occasionally develop in a normal liver, most cases are associated with long-lasting chronic liver disease (Sherlock and Dooley, 1997). Between 2.2% and 55% of autopsied cirrhotic patients have HCC, and about 80% of HCC patients have coexisting cirrhosis (Jeng and Tsai, 1991; Simonetti et al, 1991; Tsai et al, 1994*a–d*, 1996*a*; Sherlock and Dooley, 1997). Because the majority of HCC arises in the cirrhotic liver, and because cirrhosis seems to be a major determinant or promoting factor in the development of HCC, recognition of the conditions associated with HCC development can contribute to the knowledge of hepatic carcinogenesis.

*Received 4 March 1997*
*Revised 5 March 1997*
*Accepted 29 March 1997*

*Correspondence to:* Jung-Fa Tsai, Department of Internal Medicine, Kaohsiung Medical College, 100 Shih-Chuan 1 Rd, Kaohsiung, Taiwan 80708

Although most HBV- and/or HCV-infected patients develop HCC only after their disease has progressed to cirrhosis, it is unknown whether the risk of progression to HCC is different in relation to the aetiology of cirrhosis. Moreover, multiple viral infection frequently occurs in patients with chronic liver disease. For example, around 10–20% of chronic HBsAg carriers have been reported to be positive for anti-HCV, and 2–10% of anti-HCV-positive patients have been reported to have markers of HBV infection (Kaklamani et al, 1991; Simonetti et al, 1992; Tsai et al, 1993, 1994*a–g*, 1996*a,b*; Benvegnu et al, 1994; Alberti et al, 1995). It is not certain whether this co-infection increases the likelihood of the development of HCC. Our previous case–control studies have shown that there is an interacting role between HBV and HCV infection in the development of chronic hepatitis (Tsai et al, 1996*b*), cirrhosis (Tsai et al, 1993, 1994*a*), and HCC (Tsai et al, 1994*a–d*, 1996*a*). A synergistic interaction between HBV and HCV in the development of HCC has also been speculated by other investigators (Kaklamani et al, 1991; Simonetti et al, 1992). However, most previous studies have aimed to explore the association between HCV/HBV and HCC and have been limited to case-series studies (Jeng and Tsai, 1991; Kaklamani et al, 1991) or cross-sectional case–control studies (Simonetti et al, 1992; Tsai et al, 1994*a–d*, 1996*a*). Because blood samples for the detection of viral markers were collected at the time and/or after the diagnosis of HCC, these studies have left a controversy on causal inferences. Moreover, reports assessing the incidence of HCC development and its contributing factors in cirrhotic patients related to hepatitis B and C are rare. Furthermore, the mutual confounding and interactive effects between HBV and HCV infection remain to be elucidated. We have therefore conducted a prospective study to explore these problems.

### SUBJECTS AND METHODS

### Design of the study

This was a prospective study of a cohort of 400 consecutive patients with non-alcoholic cirrhosis. At inclusion in the follow-up, patients were evaluated for HBV and HCV markers, conventional liver function tests, serum α-fetoprotein (AFP) levels and Child–Pugh grades. The patients were followed up prospectively to define the incidence of HCC development in relation to HBV and HCV infection.

### Study population

During a 6-year period, 400 consecutive patients with non-alcoholic cirrhosis were prospectively followed, each for a minimum of 4 months, after clinicopathological diagnosis at entry. All patients were hospitalized or visited outpatients clinics in Kaohsiung Medical College Hospital from January 1989 to December 1994. There were 290 men and 110 women, with a mean age of 52.0 ± 11.1 (mean ± SD) years. Cirrhosis was diagnosed by liver biopsy, abdominal sonography (portal systemic shunts, splenomegaly, spotty coarse parenchyma, nodular surface and dull or round edge), biochemical evidence of parenchymal damage plus endoscopic oesophageal or gastric varices (Tsai et al, 1993, 1994*a*). Patients were classified into the three Child–Pugh grades based on their clinical status (Pugh et al, 1973). Patients with a possible association with HCC at the time of entry were excluded. Written informed consent was obtained from each subject studied. The study was approved by the Investigation and Ethics Committee of the hospital.

### Serological examination

At inclusion and during the subsequent visits, sera were taken from all patients. Hepatitis B surface antigen (HBsAg), anti-HCV and AFP were tested with Ausria-II, second-generation HCV EIA and α-feto RIABEAD (Abbott Laboratories, Chicago, IL, USA) respectively. For anti-HCV, reactive specimens were re-tested. Repeatedly reactive samples were tested with another second-generation anti-HCV immunoassay (UBI HCV EIA; United Biomedical, Lake Success, NY, USA), which incorporates synthetic peptides from the capsid and non-structural protein region as the solid-phase antigen. Only specimens reactive in all three tests were considered as anti-HCV positive. Conventional liver function tests were determined with an autoanalyser.

### Follow-up of patients

Follow-up was done by periodic abdominal ultrasound examination every 4 months and more frequently if clinically indicated. Routine follow-up studies included clinical assessment, conventional liver biochemical tests, assay for AFP and real-time ultrasonography. If a space-occupying lesion of the liver was found during follow-up and/or serum AFP was ≥ 400 ng $ml^{-1}$ without aminotransferase fluctuation, aspiration cytology or biopsy was performed. The starting time was the day of enrolment. All prognostic variables were measured on that day. Follow-up time was defined as the duration from the date of enrolment to the date of HCC development, the last contact with the patients or the end of the observation period (December 31 1994). Forty-two (10.5%) patients were lost to follow-up. Because the eventual outcomes regarding the appearance of HCC were not identified in these patients, they were dealt with as censored data in the following statistics (Harrington and Fleming, 1982).

All HCC patients were diagnosed by fine-needle aspiration cytology or biopsy. Ultrasound examination was performed with a high resolution real-time instrument (Sonolayer SSA-250A, Toshiba Corporation, Tokyo, Japan) with a 3.5-MHz convex transducer. Fine-needle biopsy was done under sonographic guidance using 22G thin needles.

### Statistical analysis

Continuous data were expressed as means ± standard deviation (mean ± SD). The difference between means of unpaired continuous variables was compared with unpaired Student's *t*-test and/or one way analysis of variance with the Tukey's test when appropriate. Fisher's exact test was used to compare differences between proportions. Relative risks with 95% confidence interval (95% CI) were used to estimate associations between exposure and disease. The Mantel extension test for trend was used to examine the dose–response relationship for the risk estimates. Kaplan–Meier's product limit survival analysis was performed to evaluate the cumulative probability of HCC development in patients during follow-up (Kaplan and Meier, 1958). Univariate analysis by the Mantel–Cox log-rank test was used to define the influence of each risk factor (Peto et al, 1974). Multivariate analysis by Cox's proportional hazards model was used to evaluate the independent roles of each factor (sex, age, AFP, HBsAg, anti-HCV, alanine aminotransferase (ALT), and Child–Pugh stage) for development of HCC (Cox, 1972). All factors found to be at least marginally associated with HCC development ($P < 0.15$) were tested by multivariate analysis. Two-tailed *P*-values and 95% CIs were given when appropriate. An alpha of 0.05 was used as the indicator of statistical significance. Data analyses were performed with the computer program BMDP/Dynamic, release 7.0 (BMDP Statistical Software, Los Angeles, CA, USA).

### RESULTS

### Clinical characteristics of patients at enrolment

At the time of enrolment, 83 (20.8%) patients had anti-HCV alone and 234 (58.5%) patients had HBsAg alone. Forty (10.0%) patients were positive for HBsAg and anti-HCV. Neither HBsAg nor anti-HCV was positive in 43 (10.8%) patients. The mean age of 123 patients with anti-HCV was greater than that of patients negative for anti-HCV (57.0 ± 9.3 vs 50.0 ± 11.4 years, $P = 0.0001$). Further analysis indicated that the mean age of patients with HBsAg alone (49.0 ± 11.3 years) was lower than that of patients with anti-HCV alone (58.8 ± 6.7 years, $P < 0.01$) or patients with HBsAg and anti-HCV (56.0 ± 9.5 years, $P < 0.01$). According to the Child–Pugh classification, grade A, B and C were noted in 282 (70.5%), 100 (25.0%) and 18 (4.5%) patients respectively. The frequency of Child–Pugh C in patients co-infected with HBV/HCV infection (15.0%, 6/40) was higher than that in patients negative for either marker (2.3%, 1 out of 43; $P = 0.044$), in patients with HBsAg alone (3.4%, 8 out of 234; $P = 0.012$) and in patients with anti-HCV alone (3.6%, 3 out of 83; $P = 0.014$). There were 122 (30.5%) patients with initial serum AFP greater than 20 ng $ml^{-1}$.

© Cancer Research Campaign 1997

Table 1 Incidence of HCC development in cirrhotic patients by the status of HBsAg and anti-HCV

| | | | Development of HCC | | |
|---|---|---|---|---|---|
| HBsAg/anti-HCV at entry | *n* | Follow-up (years) | Age (years) | *n* (%) | Annual incidence (%) |
| Negative/negative | 43 | 3.2 ± 1.7[a] | 56.3 ± 5.6 | 3 (7.0) | 2.0[d,e] |
| Positive/negative | 234 | 2.9 ± 1.8 | 52.6 ± 7.9[b,c] | 45 (19.2) | 6.6[d] |
| Negative/positive | 83 | 2.9 ± 1.3 | 62.9 ± 3.8[b] | 17 (20.5) | 7.0[e] |
| Positive/positive | 40 | 2.8 ± 1.7 | 59.7 ± 8.8[c] | 15 (37.5) | 13.3[d,e] |
| Total | 400 | 3.0 ± 1.7 | 56.2 ± 8.5 | 80 (20.0) | 6.8 |

HCC, hepatocellular carcinoma; HBsAg, hepatitis B surface antigen; anti-HCV, antibodies to hepatitis C virus. [a]Continuous data are expressed as means ± SD; [b,c]$P < 0.01$ (one-way analysis of variance with the Tukey test); [d,e]$P < 0.0001$ (Mantel extension test for trend).

Table 2 Incidence of HCC development during follow-up of 400 cirrhotic patients in relation to baseline clinical manifestations

| Parameters | Groups | No cases at risk | HCC developed | |
|---|---|---|---|---|
| | | | *n* (%) | Relative risk (95% CI) |
| Sex | Men | 290 | 65 (22.4) | 1.8 (1.0–3.5) |
| | Women | 110 | 15 (13.6) | 1.0 |
| Age (years) | ≥ 50 | 253 | 66 (26.1) | 3.4 (1.7–6.5) |
| | < 50 | 147 | 14 (9.5) | 1.0 |
| AFP (ng $ml^{-1}$) | > 20 | 122 | 36 (29.5) | 2.2 (1.3–3.8) |
| | ≤ 20 | 278 | 44 (15.8) | 1.0 |
| ALT (IU $l^{-1}$)[a] | Normal (≤ 45) | 199 | 31 (15.5) | – |
| | 1X[b] < ALT ≤ 2.5 × | 113 | 23 (22.1) | – |
| | > 2.5 × | 88 | 26 (29.5) | – |
| Child–Pugh | A | 282 | 55 (19.5) | – |
| | B | 100 | 22 (22.0) | – |
| | C | 18 | 3 (16.7) | – |
| HBsAg | Positive | 274 | 60 (21.9) | 1.5 (0.8–2.6) |
| | Negative | 126 | 20 (15.8) | 1.0 |
| Anti-HCV | Positive | 123 | 32 (26.0) | 1.6 (1.0–2.9) |
| | Negative | 277 | 48 (17.3) | 1.0 |

HCC, hepatocellular carcinoma; AFP, α-fetoprotein; ALT, alanine aminotransferase; HBsAg, hepatitis B surface antigen; Anti-HCV, antibodies to hepatitis C virus. [a]$P < 0.01$ (Mantel extension test for trend); [b]1x = 45 IU $l^{-1}$

### Development of HCC during follow-up

During a follow-up period of 1185 person–years (3.0 ± 1.7 years), HCC was developed in 80 (20.0%) patients 2.8 ± 1.5 years after entry into the study. Using the Kaplan–Meier method, the cumulative frequency of being free of HCC was found to be 83.2% at the end of the third year and 44.4% at the end of the sixth year. The calculated annual incidence of HCC development was 6.8%. The annual incidence was 2.0% in patients negative for HBsAg and anti-HCV, 6.6% in patients with HBsAg alone, 7.0% in patients with anti-HCV alone and 13.4% in patients co-infected with HBV and HCV (Table 1). There was a positive linear trend in the annual incidence of HCC among patients without either marker, patients with single viral infection and patients with dual viral infection ($P_{\text{for trend}} < 0.0001$; Table 1). Among cirrhotic patients who developed HCC, there were 65 men and 15 women. Their age ranged from 33 to 74 (56.2 ± 8.5) years. When HCC was diagnosed, 30 (37.5%) patients had serum AFP level ≤ 20 ng $ml^{-1}$, 25 (31.3%) patients had AFP level between 21 and 399 ng $ml^{-1}$ and another 25 (31.3%) patients had serum AFP greater than 400 ng $ml^{-1}$. The size of HCC at diagnosis was less than 2 cm in 26 (32.5%) patients, between 2.1 cm and 3 cm in 41 (51.2%) patients, between 3.1 cm and 4 cm in 7 (8.8%) patients and between 4.1 cm and 5.0 cm in 2 (2.5%) patients. Diffuse HCC was noted in 4 (5.0%) patients. The median diameter of HCC was 2.8 cm.

### Development of HCC in relation to baseline clinical features

As shown in Table 2, although male cirrhotic patients developed HCC more frequently than did female cirrhotic patients, the difference was not significant. The frequency of HCC development was higher in those older than 50 years ($P < 0.0001$) and in patients with serum AFP > 20 ng $ml^{-1}$ ($P < 0.003$). There is a positive linear trend in the incidence of HCC as serum ALT level increased ($P_{\text{for trend}} < 0.01$) (Table 2). Although the frequency of HCC development in HBsAg-positive (or anti-HCV-positive) cirrhotic subjects was slightly higher than HBsAg-negative (or anti-HCV-negative) cirrhotic subjects, the difference did not reach statistical significance. However, the incidence of HCC development in patients with concurrent HCV and HBV infection (37.5%) was

© Cancer Research Campaign 1997

**Figure 1** Cumulative risk for free of hepatocellular carcinoma in relation to serum alanine aminotransferase and α-fetoprotein levels, Child–Pugh grade, and age at enrolment. ALT, alanine aminotransferase; 1× = 45 IU $l^{-1}$; AFP, α-fetoprotein higher than in patients with anti-HCV alone (20.5%, $P < 0.04$), in patients with HBsAg alone (19.2%, $P < 0.04$) or in patients negative for either infection (7.0%, $P < 0.001$) (Table 1). There was a linear trend in the incidence of HCC development among patients negative for either viral marker, patients with each marker alone and those with HBsAg and anti-HCV ($P_{\text{for trend}} < 0.0001$; Table 1).

### Univariate analysis of risk factors for HCC development

As shown in Figure 1, univariate analysis indicated that the cumulative risk for HCC development was significantly higher in patients older than 50 years ($P$ = 0.0001), in those with AFP > 20 ng $ml^{-1}$ ($P$ = 0.001) and in patients with raised ALT levels at entry ($P$ = 0.0001). Compared with patients with Child-Pugh A disease, cirrhotic patients with Child–Pugh B or C have a significantly higher incidence of developing HCC ($P$ = 0.0001). The cumulative risk of being free of HCC in relation to status of HBsAg and anti-HCV is shown in Figure 2. Although patients with anti-HCV had a higher risk of HCC ($P$ = 0.025), there was no such difference regarding HBsAg status. Compared with patients with at least one viral marker, cirrhotic subjects negative for HBsAg and anti-HCV had a lower risk of HCC ($P$ = 0.014). The highest risk of HCC was noted in patients co-infected with HBV and HCV ($P$ = 0.005). There was no statistical difference in the cumulative risk of HCC with regard to sex (data not shown).

### Multivariate analysis of risk factors for HCC development

As shown in Table 3, multivariate analysis with Cox's proportional hazard model indicated that concurrent HCV and HBV infection ($P$ = 0.004), anti-HCV alone ($P$ = 0.030), HBsAg alone ($P$ = 0.020) raised ALT at entry ($P$ = 0.001 for ALT ≤ 2.5-fold upper limit of normal and $P$ = 0.0001 for ALT > 2.5-fold upper limit of normal), Child–Pugh B ($P$ = 0.0001) or Child–Pugh C ($P$ = 0.002), age ≥ 50 years ($P$ = 0.0001), and AFP > 20 ng $ml^{-1}$ ($P$ = 0.002) were independent risk factors for HCC.

### DISCUSSION

By using a formal epidemiological approach, this prospective study provides evidence that concurrent-HBV and HCV infection predisposes cirrhotic patients to a significantly higher risk of HCC development than patients with single viral infection. Furthermore, although both viruses do cause cirrhosis and eventually HCC directly or indirectly, the other factors also play some role in cancer promotion in patients with cirrhosis. Continuous necroinflammation of liver tissue, which was expressed by elevated levels of AFP and ALT, and worsening Child–Pugh grades were also significant promoting factors.

In this study, seropositivity of HBsAg and/or anti-HCV was noted in 89.2% (357 out of 400) of cirrhotic patients at enrolment and in 93% (77 out of 80) of patients with cirrhotic HCC (Table 1). The strong association between HCC development and these two viral infections indicates that HBV and HCV operate a strong oncogenic effect on liver cells. Moreover, this longitudinal study indicates that the incidence of HCC development in cirrhotic patients with HBV/HCV co-infection (25%; 15 out of 60) was higher than in those with HBsAg alone (14.5%; 40 out of 274; $P$ = 0.04). In a previous prospective study performed in an HCV hyperendemic area, concurrent HBV and HCV infection was found in 17.3% (8 out of 46) of HBsAg-positive cirrhotic patients developing HCC and in 7.7% (15 out of 195) of cirrhotic patients with HBsAg (Ikeda et al, 1993). Taiwan is an area hyperendemic for HBV infection. This study indicates that the risk for HCC in patients with HBV/HCV co-infection was also significantly higher than that in those with single viral infection in a HBV hyperendemic area (Table 1). The precise mechanisms by which dual HBV/HCV infection promotes tumour development remain to be elucidated. Our results and most epidemiological studies indicate

© Cancer Research Campaign 1997

**Figure 2** Cumulative risk for free of hepatocellular carcinoma in relation to status of hepatitis B surface antigen and antibodies to hepatitis C virus. HBsAg, hepatitis B surface antigen; anti-HCV, antibodies to hepatitis C virus

**Table 3** Multivariate analysis of factors associated with the development of HCC in cirrhotic patients by Cox's proportional hazard model

| Variables | Coefficient | s.e. | *P*-value | Hazard rate (95% CI) |
|---|---|---|---|---|
| Anti-HCV+ alone | 1.32 | 0.63 | 0.030 | 3.74 (1.07–13.07) |
| HBsAg+ alone | 1.40 | 0.60 | 0.020 | 4.06 (1.23–13.34) |
| Anti-HCV+/HBsAg+ | 1.85 | 0.64 | 0.004 | 6.41 (1.80–22.80) |
| Raised ALT (≤ 2.5x[a]) | 1.06 | 0.31 | 0.001 | 2.91 (1.58–5.36) |
| Raised ALT (> 2.5 x) | 1.53 | 0.28 | 0.0001 | 4.63 (2.65–8.09) |
| Child–Pugh B | 1.06 | 0.26 | 0.0001 | 2.90 (1.71–4.92) |
| Child–Pugh C | 2.09 | 0.66 | 0.002 | 8.08 (2.20–29.65) |
| AFP > 20 ng ml$^{-1}$ | 0.74 | 0.23 | 0.002 | 2.11 (1.32–3.37) |
| Age ≥ 50 years | 1.39 | 0.31 | 0.0001 | 4.02 (2.19–7.40) |

HCC, hepatocellular carcinoma; Anti-HCV, antibodies to hepatitis C virus; HBsAg, hepatitis B surface antigen; ALT, alanine aminotransferase; AFP, α-fetoprotein; CI, confidence interval. [a] 1x = 45 IU l$^{-1}$.

that circulating anti-HCV is present appreciably more often in HBsAg-negative than in HBsAg-positive patients with HCC (Jeng and Tsai, 1991; Kaklamani et al, 1991; Simonetti et al, 1992; Tsai et al, 1994*a–d*, 1996*a*). These observations suggest that HCV is particularly likely to be a causative factor for HCC in patients in whom HBV cannot be incriminated. The impact of HCV infection in patients with chronic HBV infection has not been well characterized. HCV infection may prolong the hepatic injury of HBV and progress to cirrhosis. Compared with patients with single HBV or HCV infection, the significantly higher frequency of patients with Child–Pugh C in our patients with cirrhosis alone indicates that dual HBV/HCV infection causes more severe liver damage. This observation confirms that liver disease in patients with multiple hepatotropic virus infection tends to be more severe than in patients with single infection (Tsai et al, 1993, 1994*a*, 1995*a,b*; Alberti et al, 1995). It is well known that superinfection of HBV in a chronic HCV carrier can cause more severe hepatitis (and/or fulminant hepatitis) and that the same holds true for chronic HBV carriers who develop a HCV superinfection (Alberti et al, 1995). Furthermore, case–control studies have indicated that dual infection with HCV and HBV has a much higher odds ratio for the development of HCC (Kaklamani et al, 1991; Simonetti et al, 1992; Tsai et al, 1994*a–d*, 1996*a*; Alberti et al, 1996). Recently, a prospective follow-up study in Italy suggested that dual HBsAg and anti-HCV positivity in patients with cirrhosis is an independent and significant determinant for the development of HCC (Benvegnu et al, 1994). In addition, several studies on HCC patients negative for seral HBsAg have indeed shown that a significant number of patients with seral anti-HCV have both HBV and HCV genomic sequences in tumorous and non-tumorous liver tissue (Paterlini et al, 1993; Diamaantis et al, 1994). On the other hand, although HCV may cause more severe liver damage than HBV (Tsai et al, 1994*e*, 1996*b*; Takano et al, 1995), our results show no difference in the incidence of HCC between the two viral infections. However, a significantly higher incidence of HCC in chronic hepatitis C than in chronic hepatitis B was reported previously, and was attributed to more severe liver damage caused by HCV infection (Takano et al, 1995).

*© Cancer Research Campaign 1997*

It is noteworthy that the mean age in HCC patients with anti-HCV alone was older than that in patients with HBsAg alone (Table 1). In populations in which HBV infection is endemic, HCC develops at a younger age in patients with HBV-related tumours than in those with HCV-related tumours (Jeng and Tsai, 1991; Tsai et al, 1994*a–d*, 1996*a*). The age difference suggests that in an HBV-endemic area persistent HCV infection is acquired later in life than is chronic HBV infection. At the time of enrolment, the mean age of our patients with anti-HCV was 7 years older than anti-HCV negative patients. Further analysis indicated that patients with HBsAg alone were significantly younger than patients with anti-HCV alone or patients with dual HBV/HCV infection. A similar trend has also been noted in patients who developed HCC (Table 1). In Taiwan the HCV carrier rate in the general population tended to be higher among older subjects (Tsai et al, 1997), and most of the HBsAg carriers had been infected perinatally or horizontally during early childhood (Chen 1993). The concurrent HBV and HCV infection in our patients might be caused by superinfection of HCV in previous chronic HBV carriers. Although HBV alone may be sufficient for development of HCC, it seems probable that HBV may also act as an 'initiating' factor through its capacity of disarranging cellular genes, whereas HCV may behave as a 'promoting' factor by causing persistent liver cell necrosis and regeneration, resulting in cirrhosis and/or HCC (Moradpour and Wand, 1996).

It is worth noting that there is an association between higher serum ALT activity and increased risk of developing HCC (Figure 1). This observation indicates that the degree of hepatic injury culminating in cirrhosis also correlates with the increased risk for HCC (Curley et al, 1995). In addition, the cumulative risk of developing HCC was statistically higher in patients with worsening Child–Pugh grades (Figure 1) (Ikeda et al, 1993; Diamantis et al, 1994). A recent study also indicated significantly higher incidence of HCC in patients with severe chronic active hepatitis and/or cirrhosis than in those with histologically less severe liver injury (Curley et al, 1995). Regardless of aetiology, chronic liver cell injury and the associated inflammatory and regenerative response constitute a preneoplastic process that will inevitably evolve towards malignancy if sufficient time is allowed to elapse (Popper, 1988; Moradpour and Wand, 1996; Sherlock and Dooley, 1997). It is reasonable to assume that persistent infection, continuous liver damage and cirrhosis are the most probable mechanisms by which HBV/HCV contribute to the pathogenesis of HCC.

The yearly incidence of HCC in cirrhotics was from 2 to 5% in the West, compared with 6–11% in the Far East (Colombo, 1995). The calculated annual incidence of HCC in this study is 6.8%. The incidence of HCC generally increases in proportion to age (Takano et al, 1995). Our result also indicates that age greater than 50 years is an independent risk factor for HCC (Table 2 and Figure 1). Age may be either a determinant in itself or simply a reflection of the duration of the liver disease (Simonetti et al, 1991). There is evidence that HCC risk shows a linear increase during a patient's lifetime (Takano et al, 1995). Consistent with this fact is the observation that the cumulative probability of developing HCC gradually increases during follow-up. In addition, a high AFP value at enrolment was also a predictor for HCC (Table 2 and Figure 1). A similar observation has also been reported previously (Tanano et al, 1995; Sherlock and Dooley 1997). Although the mechanism of the increase in serum AFP level in benign liver disease is complex and not understood, this increase has been related to more aggressive histological activity in chronic liver disease (Collazos et al, 1992). This observation supports the hypothesis that more severe hepatic inflammation is a risk factor for liver cancer.

In conclusion, we have shown frequent coexistence of HBV and HCV markers in Chinese patients with cirrhosis and/or cirrhotic HCC. We think that infection with HBV and HCV increases the risk of more severe liver disease than infection with single viral infection. We also provide evidence of the independent role and additive interaction of HBV and HCV infection on the development of HCC. Equally important is the fact that the degree of hepatic injury culminating in cirrhosis also correlates with the increased risk of HCC. The results of this and other studies indicate that patients with HBV/HCV co-infection should be monitored with great care for HCC development. Adequate treatment of chronic HBV/HCV infection may decrease risk of HCC development.

### ACKNOWLEDGEMENT

This study was supported in part by a grant from the National Science Council of the Republic of China (NSC 85-2331-B-037-084 M14).

### REFERENCES

Alberti A, Pontisso P, Chemello L, Fattovich G, Benvegnu L, Belussi F and De Mitri MS (1995) The interaction between hepatitis B and hepatitis C virus in acute and chronic liver disease. *J Hepatol* **22 (suppl.)**: 38–41

Benvegnu L, Fattovich G, Noventa F, Tremolada F, Chemello L, Cecchetto A and Alberti A (1994) Concurrent hepatitis B and C virus infection and risk of hepatocellular carcinoma in cirrhosis. A prospective study. *Cancer* **74**: 2442–2448

Chen DS (1993) Natural history of chronic hepatitis B virus infection: new light on an old story. *J Gastroenterol Hepatol* **8**: 470–475

Collazos J, Genolla J and Ruibal A (1992) Preliminary study of alpha-fetoprotein in nonmalignant liver disease: a clinico-biochemical evaluation. *Int J Biol Markers* **7**: 97–102

Colombo M (1995) Should patients with chronic viral hepatitis be screened for hepatocellular carcinoma? *Viral Hepatitis Rev* **1**: 67–75

Cox DR (1972) Regression models and life tables. *J R Stat Soc* **34** (Series B): 187–220

Curley SA, Izzo F, Gallipoli A, de Bellis M, Cremona F and Parisi V (1995) Identification and screening of 416 patients with chronic hepatitis at high risk to develop hepatocellular cancer. *Ann Surg* **222**: 375–380

Diamantis ID, McGandy CE, Chen TJ, Liaw YF, Gudat F and Bianchi L (1994) Detection of hepatitis B and C viruses in liver tissue with hepatocellular carcinoma. *J Hepatol* **20**: 405–409

Harrington DP and Fleming TR (1982) A class of rank test procedures for censored survival data. *Biometrica* **69**: 553–566

Ikeda K, Saitoh S, Koida I, Arase Y, Tsubota A, Chayama K, Kimada H and Kawanishi M (1993) Multivariate analysis of risk factors for hepatocellular carcinogenesis: A prospective observation of 795 patients with viral and alcoholic cirrhosis. *Hepatology* **18**: 47–53

Jeng JE and Tsai JF (1991) Hepatitis C virus antibody in hepatocellular carcinoma in Taiwan. *J Med Virol* **34**: 74–77

Kaklamani E, Trichopoulos D, Tzonou A, Zavitsanos X, Koumantaki Y, Hatzakis A, Hsieh CC and Hatziyannis S (1991) Hepatitis B and C viruses and their interaction in the origin of hepatocellular carcinoma. *JAMA* **265**: 1974–1976

Kaplan EL and Meier P (1958) Nonparametric estimation from incomplete observation. *Am Stat Assoc* **53**: 457–458

Kiyosawa K, Sodeyama T, Tanaka E, Gibo Y, Yoshizawa K, Nakano Y, Furuta S, Akahane Y, Nishioka K, Purcell RH and Alter HJ (1990) Interrelationship of blood transfusion, non-A, non-B hepatitis and hepatocellular carcinoma: analysis by detection of antibody to hepatitis C virus. *Hepatology* **12**: 671–675

Moradpour D and Wand JR (1996) Hepatic oncogenesis. In *Hepatology: a Textbook of Liver Disease*, 3rd edn, Zakim D and Boyer TD (eds) pp. 1490–1512. W.B. Saunders: Philadelphia

Paterlini P, Driss F, Nalpas B, Pisi E, Franco D, Berthelot P and Brechot C (1993) Persistence of hepatitis B and C viral genomes in primary liver cancers from HBsAg-negative patients: a study of low endemic area. *Hepatology* **17**: 20–29

© Cancer Research Campaign 1997 

Peto R, Pike MC, Armitage P, Breslow NE, Cox DR, Howard SV, Mantel N, McPherson K, Peto J and Smith PG (1974) Design and analysis of randomized clinical trials requiring prolonged observation of each patient. *Br J Cancer* **34**: 585–612

Popper H (1988) Viral versus chemical hepatocarcinogenesis. *J Hepatol* **6**: 229–238

Pugh RN, Murray-Lyon IM, Dawson JL, Peitroni MC and Williams R (1973) Transection of the oesophagus for bleeding oesophageal varices. *Br J Surg* **60**: 646–649

Sherlock S and Dooley J (1997) Hepatic tumours. In *Disease of the Liver and Biliary System*, 10th edn, pp. 531–559. Blackwell Science: Oxford

Simonetti RG, Camma C, Fiorello F, Politi F, D'Amico G and Pagliaro L (1991) Hepatocellular carcinoma: a worldwide problem and the major risk factors. *Dig Dis Sci* **36**: 962–972

Simonetti RG, Camma C, Fiorella F, Cottone M, Rapicetti M, Marino L, Fiorentino G, Craxia A, Ciccaglione A, Giuseppetti R, Stroffolini T and Pagliaro L (1992) Hepatitis C virus infection as a risk factor for hepatocellular carcinoma in patients with cirrhosis. *Ann Intern Med* **116**: 97–102

Takano S, Yokosuka O, Imazeki F, Tagawa M and Omata M (1995) Incidence of hepatocellular carcinoma in chronic hepatitis B and C: a prospective study of 251 patients. *Hepatology* **21**: 650–655

Tong MJ, el-Farra NS, Reikes AR and Co RL (1995) Clinical outcomes after transfusion-associated hepatitis C. *N Engl J Med* **332**: 1463–1466

Tsai JF, Chang WY, Jeng JE, Ho MS, Wang LY, Hsieh MY, Chen SC, Chuang WL, Lin ZY and Tsai JH (1993) Hepatitis C virus infection as a risk factor for non-alcoholic liver cirrhosis in Taiwan. *J Med Virol* **41**: 296–300

Tsai JF, Chang WY, Jeng JE, Ho MS, Lin ZY and Tsai JH (1994*a*) Hepatitis B and C virus infection as risk factors for liver cirrhosis and cirrhotic hepatocellular carcinoma: a case–control study. *Liver* **14**: 98–102

Tsai JF, Chang WY, Jeng JE, Ho MS, Lin ZY and Tsai JH (1994*b*) Frequency of raised α-fetoprotein level among Chinese patients with hepatocellular carcinoma related to hepatitis B and C. *Br J Cancer* **69**: 1157–1159

Tsai JF, Chang WY, Jeng JE, Ho MS, Lin ZY and Tsai JH (1994*c*) Effects of hepatitis C and B viruses infection on the development of hepatocellular carcinoma. *J Med Virol* **44**: 92–95

Tsai JF, Jeng JE, Ho MS, Chang WY, Lin ZY and Tsai JH (1994*d*) Hepatitis B and C virus infection as risk factors for hepatocellular carcinoma in Chinese: a case–control study. *Int J Cancer* **56**: 619–621

Tsai JF, Jeng JE, Chang WY, Ho MS, Lin ZY and Tsai JH (1994*e*) Hepatitis C virus infection among patients with chronic liver disease in an area hyperendemic for hepatitis B. *Scand J Gastroenterol* **29**: 550–552

Tsai JF, Jeng JE, Chang WY, Lin ZY and Tsai JH (1994*f*) Antibodies to hepatitis E virus among Chinese patients with acute hepatitis in Taiwan. *J Med Virol* **43**: 341–344

Tsai JF, Jeng JE, Chang WY, Lin ZY and Tsai JH (1994*g*) Antibodies to hepatitis E and A viruses among patients with non-alcoholic chronic liver disease in Taiwan. *Scand J Gastroenterol* **29**: 651–654

Tsai JF, Jeng JE, Chang WY, Ho MS, Lin ZY and Tsai JH (1995*a*) Increased IgM-containing circulating immune complexes in patients co-infected with hepatitis C and hepatitis B. *Medicine* **74**: 136–143

Tsai JF, Jeng JE, Chang WY, Ho MS, Lin ZY and Tsai JH (1995*b*) Circulating immune complexes in chronic hepatitis related to hepatitis C and B viruses infection. *Clin Immunol Immunopathol* **75**: 39–44

Tsai JF, Jeng JE, Ho MS, Chang WY, Hsieh MY, Lin ZY and Tsai JH (1996*a*) Additive effect modification of hepatitis B surface antigen and e antigen on the development of hepatocellular carcinoma. *Br J Cancer* **73**: 1498–1502

Tsai JF, Jeng JE, Ho MS, Chang WY, Hsieh MY, Lin ZY and Tsai JH (1996*b*) Independent and additive effect modification of hepatitis C and B viruses infection on the development of chronic hepatitis. *J Hepatol* **24**: 271–276

Tsai JF, Jeng JE, Ho MS, Wang CS, Chang WY, Hsieh MY, Lin ZY and Tsai JH (1997) Serum alanine aminotransferase level in relation to hepatitis B and C viruses infection among blood donors. *Liver* **17**: 24–29

© Cancer Research Campaign 1997